# In Vitro Enhanced Bonding of Silane‐Modified Adhesive Systems in Fiber Post Cementation

**DOI:** 10.1155/ijod/5593764

**Published:** 2025-09-27

**Authors:** Thais Pantoja de França, João Victor Frazão Câmara, Leonardo Queiroz Athias, Renata Antoun Simão, Maíra Prado

**Affiliations:** ^1^ Postgraduate Program in Dentistry, Veiga de Almeida University, Rio de Janeiro, RJ, Brazil, uva.br; ^2^ Saarland University, Clinic of Operative Dentistry, Periodontology and Preventive Dentistry, Homburg, Saarland, Germany, uni-saarland.de; ^3^ Brazilian Institute of Geography and Statistics, Rio de Janeiro, RJ, Brazil; ^4^ Department of Materials Science and Engineering, Federal University of Rio de Janeiro, Rio de Janeiro, RJ, Brazil, ufrj.br

**Keywords:** adhesives, bond strength, silane

## Abstract

**Objective:**

To compare the effect of applying silane prior to the adhesive with an adhesive containing silane in its composition in the cementation of fiber posts to conventional resin cement.

**Materials and Methods:**

In total 20 fiber posts were divided into two groups: silane/adhesive (G1): application of silane, followed by the application of the adhesive; and adhesive with silane (G2): application of adhesive containing silane in its composition. The test specimens were obtained from cylinders of AllCem Core resin cement, with the fiber postpositioned in the center of its long axis. The post/cement assembly was sectioned, and evaluated for bond strength (BS) by pushout test, and fracture pattern was analyzed with a stereoscopic microscope.

**Results:**

No significant difference in BS was observed between G1 (silane + adhesive) and G2 (adhesive containing silane). For fracture patterns, Type 3 (cohesive post and cement) was the most observed.

**Conclusions:**

The use of an adhesive containing silane, as per the adopted protocol, did not compromise BS when fiber posts were cemented with Allcem CORE cement. Both techniques exhibited similar results, with minor differences in fracture patterns.

## 1. Introduction

Coronary infiltration is one of the causes of failure in endodontics [1]. Therefore, endodontic treatment can only be considered complete when the definitive restoration of the access cavity is performed. Thus, to ensure predictability of success, it is important that this restoration is done immediately after the endodontic treatment is completed [2].

Glass fiber posts are an alternative to traditional metal cores in the restoration of endodontically treated teeth [3]. They have some advantages, such as aesthetics and a modulus of elasticity similar to that of dentin, which reduces intraradicular stress and the likelihood of fracture. Additionally, they can be cemented in a single session, immediately after the end of the endodontic treatment. They also have the ability to bond to resin cement, which in turn bonds to dentin through adhesive techniques [3–5].

Failures in the adhesive process can compromise the success of restorative and endodontic treatment. It is known that the main failure in the bonding of fiber posts occurs at the cement/dentin interface, due to the complexity and sensitivity of the adhesive and cementation technique. Thus, simplifying the cementation protocol with the use of universal adhesives or self‐adhesive cements aims to eliminate critical steps in the bonding process, such as the application of phosphoric acid, while also reducing treatment time [5–7].

Such failures can also occur at the composite/post interface. To prevent these failures, several modifications to the post surface have been proposed, including chemical and mechanical treatments such as the application of silane, hydrofluoric acid, phosphoric acid, hydrogen peroxide, and aluminum oxide particle blasting [8–12]. Recently, with the aim of simplifying the technique and improving adhesion, silane was incorporated into the composition of some adhesives. The addition of silane to adhesive systems could promote effective chemical bonding between inorganic surfaces and the organic matrix of adhesives or composite resins. Silane is a bifunctional coupling agent, possessing functional groups that can bond to both the inorganic phase (e.g., silica) and the organic phase (e.g., methacrylates) [13].

However, to date, there are no reports in the literature comparing the effect of these new materials to the classical process of post‐silanization followed by adhesive application. Thus, the objective of the present study was to compare the effect of applying silane prior to the adhesive with an adhesive containing silane in its composition in the cementation of fiber posts to a conventional resin cement. The null hypothesis to be tested is that there is no significant difference in bond strength (BS) between the application of silane prior to the adhesive and the use of an adhesive containing silane.

## 2. Materials and Methods

### 2.1. Sample Size

The sample size was estimated based on a previous study (Prado et al.) [14]. The ANOVA test from the F‐test family was selected in the G Power program (3.1.7; Heinrich Heine University Düsseldorf). Thus, for analysis with a significance level of 5%, a test power of 95%, and considering an effect size ( = 1.13), a total of 10 samples (*n* = 5 per group) was indicated as the ideal sample size to observe a statistical difference.

### 2.2. Experimental Groups

Twenty white post DC3 fiber posts (FGM, Joinville, SC, Brazil) were used. The posts were cleaned with 70% alcohol to remove contaminants [3]. Subsequently, the posts were randomly divided into two groups: (G1): application of silane, followed by the application of the adhesive; adhesive with silane (G2): application of the adhesive containing silane in its composition.

### 2.3. Preparation of the Test Specimens and Protocol

The test specimens for the pushout test were obtained from resin cement cylinders containing the fiber post, positioned at the center of its long axis. For this, a cylindrical silicone mold of 10 mm in height and 6 mm in internal diameter was used, made with the Transparent Addition Silicone Silic‐One Clear Body I (FGM, Joinville, SC, Brazil).

In G1, the Prosil silane (FGM, Joinville, SC, Brazil) was applied on the postsurface and wait 1 min, followed by the application of the Ambar Universal APS adhesive (FGM, Joinville, SC, Brazil), and its polymerization for 20 s using the Quazar light‐curing unit (1230 mW/cm [2] ‐ FGM, Joinville, SC, Brazil) prior to its insertion into the mold. In G2, the adhesive containing silane, Ambar Universal APS Plus (FGM, Joinville, SC, Brazil), was applied and photoactivated by 20 s.

The bottom of the mold was fixed to a transparent adhesive tape on a flat surface. Then, the post was positioned with the help of clinical tweezers, with the coronal (cylindrical) end fixed to the adhesive tape, at the center of the mold, parallel to it. The tweezers did not make contact with the cylindrical part of the post in order to avoid contamination of the sample. The posts were positioned at the center of the mold, and the mold was filled with Allcem Core cement (FGM, Joinville, SC, Brazil). The cement was polymerized for 40 s on the upper and inner faces and for 10 seconds on each lateral face. The post/resin cement assembly was removed from the mold and stored at 37°C for 24 h.

### 2.4. Pushout Analysis

The post/resin cement assembly was positioned in a cutting machine (Isomet, Buehler, Lake Bluff, IL, USA) perpendicular to the diamond disc (Series 15HC, Buehler, Lake Bluff, IL, USA), in order to produce slices of approximately 1 mm in thickness. The exact thickness of each slice was determined using a digital caliper (MPI/E‐101, Mytutoyo, Tokyo, Japan). A total of four discs were analyzed per sample, totaling 40 discs per group.

**Figure 1:**
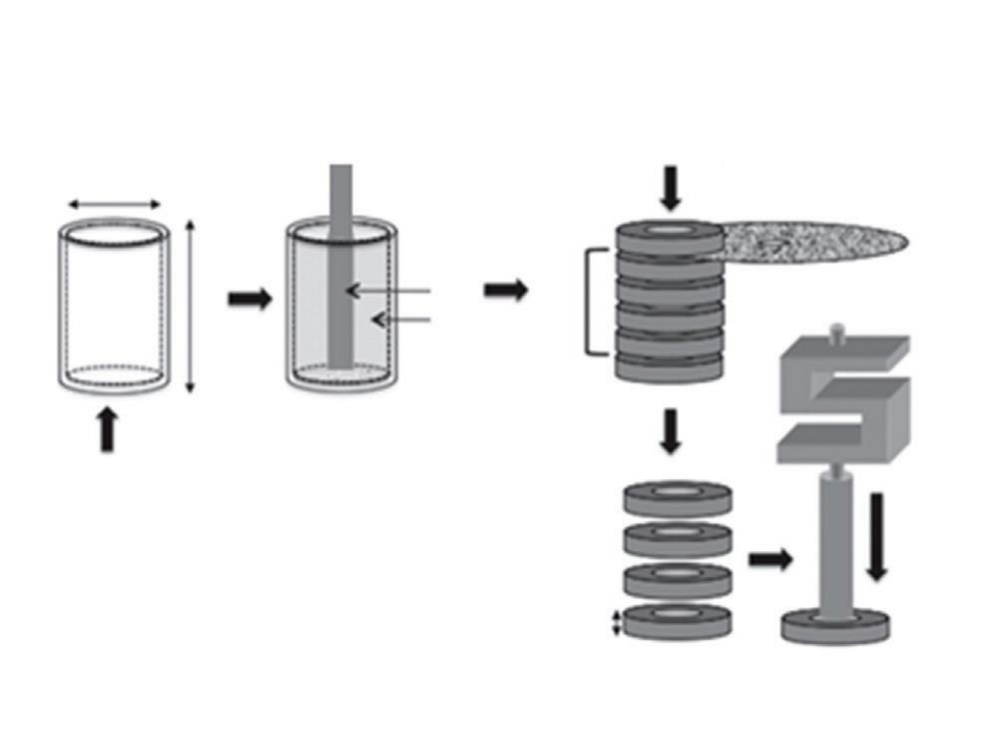
Schematic drawing illustrating the preparation of the test specimens (Prado et al.)[14].

The samples were positioned on a stainless‐steel metal base with a central hole, with the postpositioned in the direction of the hole (Figure 1). The assembly was positioned on the base of the universal testing machine EMIC DL1000 (São José dos Pinhais, PR, Brazil) with a load of 20 kgf. A metal rod with an active tip of 1.2 mm in diameter was fixed in the machine’s gripping device and positioned at the center of the fiber post.

The shear strength extrusion (pushout) test was conducted at a speed of 0.5 mm/min. The BS values were obtained in newtons (N). To express the BS in megapascals (MPa), the recorded load value in newtons (N) will be divided by the area (mm^2^) of the adhesive interface, using the following formula:

A=2 πrh,

where *π* = 3.14; *r* = radius of the post; *h* = thickness of the slice, in millimeters.

### 2.5. Fracture Pattern Analysis

After fracture, the samples were analyzed with a stereoscopic microscope (Carl Zeiss Microscopia GmbH, Munich, Germany) to determine the fracture pattern as follows: type 1: cohesive in post; type 2: cohesive in cement; type 3: cohesive post and cement; type 4: adhesive post/cement; and type 5: mixed (association between cohesive and adhesive).

### 2.6. Statistical Analysis

The BS data were tabulated and subjected to the Kolmogorov–Smirnov normality test (*α* = 0.05). The data were statistically analyzed using Mann–Whitney test with a significance level of 5%.

## 3. Results

**Figure 2:**
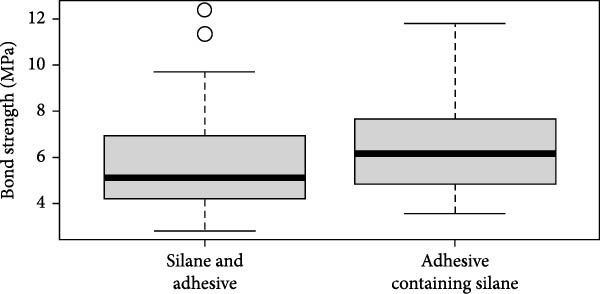
Box plot of bond strength according to the different treatments. (G1) Conventional technique of silane application, followed by adhesive and light curing and (G2) adhesive containing silane. Horizontal bars in the boxes represent the median for each group. Data were analyzed by Mann–Whitney test (*p* =  0.053, *n* = 10 post/group—40 slices/group).

**Figure 3:**
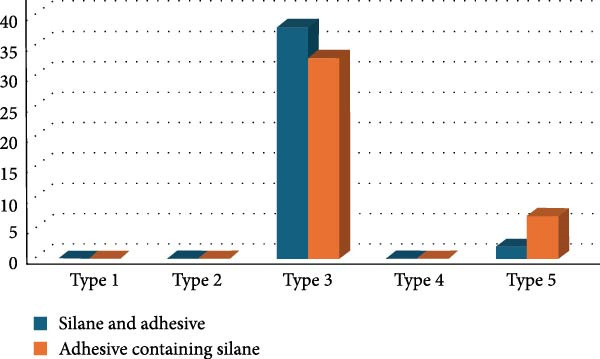
Fracture pattern of silane and adhesive (blue bars) and adhesive containing silane (orange bars).

Figure 2 illustrates the median for the different groups. No significant difference between the conventional technique (G1) and the tested adhesive (G2) in relation BS was observed. Regarding the fracture pattern (Figure 3), type 3 was the most observed, with Silane and adhesive outperforming the Adhesive containing silane. Type 5 shows only a small difference between the two groups, with the Adhesive containing silane slightly outperforming the Silane and adhesive. Type 1, type 2, and type 4 were not observed in both groups.

## 4. Discussion

The application of silane before adhesive bonding is a well‐established procedure in fields such as dentistry and materials science, particularly for enhancing the bond between inorganic materials (e.g., fiber glass post) and organic adhesives [15]. Silane acts as a coupling agent that facilitates the interaction between these materials, improving the strength and durability of the bond. Silane molecules contain both reactive silanol groups, which bond to inorganic surfaces like metal or ceramic, and alkoxy groups, which can form bonds with organic adhesives. When applied, the silane reacts with the surface to create a stable siloxane bond, improving the surface energy and wettability. This, in turn, enhances the adhesive’s ability to form a strong and durable bond [16].

The primary advantage of applying silane before the adhesive is the improved BS between the post and the adhesive. The silane treatment promotes a chemical bond at the interface, significantly enhancing the adhesion between the surface and the organic adhesive. This process also contributes to the long‐term durability of the bond, especially in environments that are exposed to moisture, such as the oral cavity or other humid conditions [17]. Additionally, silane treatment improves the wettability of the surface, allowing the adhesive to spread more evenly across the post and ensuring better contact between the adhesive and the substrate [18].

However, there are several potential disadvantages to this process. One significant drawback is the added complexity and time required for the application of silane. The additional step can increase the overall time for preparation and bonding, reducing clinical efficiency. Furthermore, if the silane is not properly applied, it can lead to surface contamination, which negatively affects the adhesive bond. Overapplication or improper drying of the silane layer can interfere with the bonding process, resulting in inconsistent BS [19]. Additionally, silane is sensitive to moisture and may undergo hydrolysis under humid conditions, which can decrease its effectiveness. As such, the application process must be carried out in a dry, controlled environment to ensure the stability of the silane layer [20].

Therefore, in response to the challenges and limitations associated with the traditional application of silane before adhesive bonding, the development of adhesives that already contain silane has gained attention in the field of materials science. In this sense, our research group showed this innovative approach aims to streamline the bonding process by integrating the silane coupling agent directly into the adhesive formulation did not affect the BS when compared to the traditional two‐step process. The production of adhesives containing silane eliminates the need for the additional step of applying silane separately, which can be time‐consuming and prone to errors.

One of the primary advantages of using an adhesive that already contains silane is the simplification of the bonding process. By combining the silane and adhesive in a single product, the need for multiple application steps is eliminated, reducing the risk of surface contamination and application inconsistencies. This results in a more efficient procedure, particularly in clinical settings where time and precision are crucial. Furthermore, this integrated approach reduces the potential for human error in the application of silane, ensuring a more uniform and consistent bond between the substrate and the adhesive. Another advantage of adhesives containing silane is the enhanced stability and reliability of the bond. Since the silane is already incorporated into the adhesive matrix, it is less susceptible to the issues of hydrolysis and moisture sensitivity that can affect silane‐treated surfaces. This provides a more stable bond in humid environments, such as the oral cavity, where traditional silane application can be compromised by moisture exposure. The silane molecules within the adhesive are specifically designed to work in synergy with the adhesive components, ensuring optimal performance and longevity of the bond [16].

Thus, the present protocol uses the conventional technique in comparison with the adhesive containing silane. Regarding the BS, there was no significant difference (*p*‐value = 0.053) between the tested groups. It could be highlighted that, even though this ⁠*p*‐value was not statistically significant, it indicates a slight trend toward a difference and should be tested in further studies. The lack of a statistically significant difference between the silane + adhesive method and the adhesive containing silane can be explained by the similar chemical mechanisms involved in both processes. In the traditional approach, silane is applied separately to the substrate, forming a siloxane layer that reacts with hydroxyl groups on the surface, enhancing the adhesive’s bonding. The adhesive then forms covalent bonds with the silanized surface. In contrast, the adhesive containing silane integrates the silane within its matrix, which allows for the same chemical reaction to occur during application, forming a siloxane layer directly on the substrate. Both methods ultimately rely on the polymerization of functional groups in the adhesive with the silanized surface, resulting in a stable and durable bond. Thus, despite the difference in application, both methods achieve comparable bonding performance, explaining the lack of statistical difference between them. Our results do not corroborate the study by Silva et al. [18], in which it was reported that using universal silane‐containing adhesive improved the repair BS of composite resin compared to two‐step etch‐and‐rinse adhesive. However, this study used blocks of nanohybrid composite resin [18]. It is important to highlight that regarding adhesion analysis, the predominant failure pattern in both groups was type 3, characterized by cohesive failure within both the post and the cement. This consistent pattern suggests that an effective bond was achieved at the postcement interface. The cohesive nature of the failure indicates that the interface itself remained intact under stress, with failure occurring within the bulk materials rather than at their junction, highlighting the strength and reliability of the adhesion between the components.

For future studies, beyond testing with increased sample sizes to increase the robustness of the results, it would be beneficial to explore the long‐term durability of the adhesive bond, particularly under different environmental conditions, such as thermal cycling or mechanical loading. Additionally, evaluating the performance of the silane‐containing adhesive in different types of resin cements and comparing it with newer adhesive technologies could provide valuable insights. Future research could also focus on microscopic analysis of the interface between the fiber posts and resin cement to assess any potential microstructural changes overtime.

## 5. Conclusion

According to the protocol adopted, the simplification of the technique, with the use of an adhesive containing silane in its composition, did not negatively affect the adhesion values when fiber posts were cemented with the conventional cement Allcem CORE.

## Disclosure

The authors do not have any financial interest in the companies whose materials are included in this article.

## Conflicts of Interest

The authors declare no conflicts of interest.

## Funding

No funding was received for this manuscript. Open Access funding enabled and organized by Projekt DEAL.

## Data Availability

The data used in this study are available from the corresponding author upon reasonable request.
